# Cytometry of chromatin bound Mcm6 and PCNA identifies two states in G1 that are separated functionally by the G1 restriction point^1^

**DOI:** 10.1186/1471-2121-11-26

**Published:** 2010-04-16

**Authors:** Phyllis S Frisa, James W Jacobberger

**Affiliations:** 1 Case Comprehensive Cancer Center, Case Western Reserve University, Cleveland, OH 44106, USA

## Abstract

**Background:**

Cytometric measurements of DNA content and chromatin-bound Mcm2 have demonstrated bimodal patterns of expression in G1. These patterns, the replication licensing function of Mcm proteins, and a correlation between Mcm loading and cell cycle commitment for cells re-entering the cell cycle, led us to test the idea that cells expressing a defined high level of chromatin-bound Mcm6 in G1 are committed - i.e., past the G1 restriction point. We developed a cell-based assay for tightly-bound PCNA (PCNA*) and Mcm6 (Mcm6*), DNA content, and a mitotic marker to clearly define G1, S, G2, and M phases of the cell cycle. hTERT-BJ1, hTERT-RPE-1, and Molt4 cells were extracted with Triton X-100 followed by methanol fixation, stained with antibodies and DAPI, then measured by cytometry.

**Results:**

Bivariate analysis of cytometric data demonstrated complex patterns with distinct clustering for all combinations of the 4 variables. In G1, cells clustered in two groups characterized by low and high Mcm6* expression. Serum starvation and release experiments showed that residence in the high group was in late G1, just prior to S phase. Kinetic experiments, employing serum withdrawal, and stathmokinetic analysis with aphidicolin, mimosine or nocodazole demonstrated that cells with high levels of Mcm6* cycled with the committed phases of the cell cycle (S, G2, and M).

**Conclusions:**

A multivariate assay for Mcm6*, PCNA*, DNA content, and a mitotic marker provides analysis capable of estimating the fraction of pre and post-restriction point G1 cells and supports the idea that there are at least two states in G1 defined by levels of chromatin bound Mcm proteins.

## Background

We have been interested in multi-variate cytometry of the mammalian somatic cell cycle. The cell cycle can be analytically divided into a variable number of states based on the correlated levels of biochemical activities provided that the level of each activity changes in a repeating pattern and that the correlated activity patterns are not identical. For the simplest case of any single variable there are essentially 5 states to consider per oscillation: initial basal level (1), increase or decrease from basal (2), maximum or minimum (max, min) (3), and decrease or increase (4) to final basal level (5), which is not biologically equivalent to initial basal. For example, the expression of cyclins E1, A2, and B1 oscillate once per cycle, out of phase with each other. All are at "initial basal" in early G1; begin expression at different times and rise at different rates; reach max at G1/S (E1) or G2/M (A2, B1); decrease in S (E1), prometaphase (A2), or anaphase (B1); are resident at "final basal" approximately in G2 (E1), metaphase (A2), or telophase (B1) [1-4]. These expression patterns are readily discerned by immunofluorescence coupled with DNA content in flow cytometry assays [5]. Thus, measurement of the cyclins, DNA, and a mitotic marker constitute highly informative analyses of cell cycle transition states characteristic of a specific population of somatic cells [e.g., [6-8]].

Because mitosis is characterized by abrupt, specific, sequentially timed proteolysis of substrates of the anaphase promoting complex/cyclosome (APC/C) [4] and an abrupt increase in kinase activities and phosphorylation of many substrates, a multi-variate cell-based approach to cell cycle analysis subdivides G2 and M in a straightforward manner [e.g., [8]]. G1 is also characterized by oscillating activities, but has not been analyzed cytometrically in the same manner as M. Here, we wished to extend to G1 this type of multi-variate analysis. One fundamental G1 sub-division is the kinetically defined uncommitted and committed states. The transition between these states has been labeled "R" (restriction point) by Yen and Pardee [9]. The exact biochemical concept or nature of R is unknown, although most investigators would agree with a complex model that integrates signaling (growth and nutrition factors; cell-substrate attachment; cell-cell contact, and cell damage) at a modular level, with an "R" module containing at least the activities of D cyclin/Cdk complexes and the Rb/E2F family of transcription factors [10-14]. For a mathematical model that formally integrates a large body of information, see Novak and Tyson [15]. For a dissenting view see [16].

Without an exact biochemical definition of "R", it may be possible to quantify cells that are in a pre-R or post-R state (~G1-pm and G1ps subphases of Zetterberg, [14]) based on measurements of specific Rb family phosphorylations; the levels of E2F transcription factors that are bound to promoters and not bound by Rb family proteins, and the levels of D and E cyclins. Previous work by Juan et al. has shown the feasibility of this approach with an antibody that binds "hypo-phosphorylated" Rb [7]. At this time, a comprehensive analysis would be difficult to achieve since there are 3 Rb family proteins, three D cyclins using 2 cyclin dependent kinases (Cdk4, Cdk6), two E cyclins using Cdk2, and 8 E2F and 2 DP protein subunits that constitute E2F transcription factors as well as at least 5 protein inhibitors of G1 Cdks. Since much of this operates as a function of specific phosphorylations, this might be rendered simpler with probes to specific phosphorylated sites on a subset of these molecules. However, these probes are not generally available and may or may not function well in cytometric assays [e.g., see [8]].

In place of the more intricate and sophisticated approach outlined above, we have asked whether the bimodal distributions shown by bivariate analyses of chromatin-bound minichromosome maintenance (Mcm) proteins and DNA content marks pre- and post-R G1 cells. These patterns were first shown by Friedrich and co-workers [17] and are based on measuring the residue of Mcm proteins that are left behind after detergent extraction. In support of this idea, Mukherjee at al. have correlated the committed period of G1 with high levels of bound Mcm protein, hyper-phosphorylated Rb, and cyclin E expression [18]. The approach employed by Freidrich et al. (extraction then fixation) was first described by Kurki et al. [19] for detecting S phase specific 'tightly' bound PCNA that was a subset of total PCNA detected by denaturing fixation. The logic is that extraction of live cells with detergent depletes cells of 'loosely' bound proteins and other molecules that can diffuse out, leaving large structures and tightly bound proteins, including those bound to chromatin. This is also close to the standard methods of creating chromatin pellets in the primary research on replication complexes.

Normal mammalian cell replication involves mechanisms to bias faithful duplication of the genome once per mitotic cell cycle. The sub-system responsible for preventing re-replication consists of a protein complex that is built on origins-of-replication (ORI) and "licensed" for initiation of DNA synthesis. A licensed pre-replication complex (pre-RC) is built on existing complexes of ORC proteins by sequential binding of Cdc6 then Cdt1/RFL-B, which then "loads" Mcm proteins. Licensing is complete after the 6 Mcm proteins are loaded onto chromatin as a functional but inactive hexameric complex. Cdt1 is rate limiting for this process. In replicating cells, licensing occurs as a continuous process from late mitosis, after loss of mitotic Cdk activity, through G1 [20,21].

In higher eukaryotes somatic cells, the rates of licensing are not known, however, one model for actively dividing cells (based on a zone within the Chinese hamster DHFR locus) suggests that licensing begins in mitosis but origin site specification occurs in G1 [22]. Models for cell cycle re-entry suggest that quiescent cells lack bound Cdc6 and Mcm proteins and therefore license in late G1 [e.g., [18,23]]. On cell cycle re-entry, Mcm loading appears to be dependent on cyclin E and Cdc7 expression and activities [23], which peak late in G1. The rate-limiting step for initiation may be unwinding of DNA by the helicase activity of the complex composed of Mcm2 through 7 [24]. Once DNA synthesis has been initiated, irrevocable rescinding of the license occurs by several mechanisms, including removal of Mcm proteins from the chromatin, that prevent reassembly of pre-RC in S phase. Reloading does not occur until the licensing process resumes in mitosis in cycling cells or in G1 in stimulated quiescent cells. This system has been reviewed in detail [e.g., see [20,21,24]].

The reported cytometric pattern of Mcm2 and DNA content for CV-1 cells provides a visualization of Mcm binding in G1 and Mcm removal from chromatin during S phase [17]. The expression of detergent resistant Mcm2 was bimodal in G1 with low and high expressing cell clusters. The high expression cluster level was coincident with the G1/S border, suggesting that cells enter S phase only after the bound Mcm complexes have reached a maximum level (max). In the published patterns, the levels in S decreased to a minimum at the S/G2 border consistent with the known cell cycle related expression [20,21,24,25].

Since origins of replication are not well mapped in higher eukaryotes, the timing of licensing and the rate of Mcm loading in actively dividing cells has not been comprehensively studied. The work of Friedrich et al. suggests that G1 cells cluster into two distinct groups with the highest cluster ~10 fold greater than the lower cluster. Since the frequency of cells existing in each cluster is proportional to the time spent at that state, CV-1 cells may pass through two G1 loading periods with a rapid transition between the two states. However, this type of pattern would also be consistent with exponential loading. While over-interpretation is not useful, the quantitative bimodality of G1 cells, if substantiated, creates a biochemically related division of G1 that could be the beginning of subdividing G1 into meaningful biochemical states that would increase the power of multi-variate analyses of the cell cycle.

Since the simplest interpretation of the CV-1 G1 patterns are an early and late subdivision, our goals were to 1) determine whether the same pattern exists in human somatic cells, and 2) determine whether the low and high clusters correlate with pre- and post-restriction point states. The second goal is an experimentally more complicated approach to verifying the early → late sequence, but if true, has the value of giving the measurement of tightly bound Mcm proteins in G1 two meanings. The first is as a measure of the functional state of the Mcm loading sub-system within the cellular/environmental context, and the second is as a surrogate marker for commitment to cell cycle progression.

Because a bivariate analysis of any immunoreactivity versus DNA content leaves an uncertainty at the G1/S interface proportional to the coefficient of variation (CV) of the two measures, we enhanced the probability that cells would be identified as either G1 or S by co-staining for tightly bound PCNA. S phase is associated with the release of the Mcm proteins and PCNA binding to replication forks (reviewed in: [26-28]). Tightly bound PCNA has also been shown by cytometry to correlate with incorporation of BrdU [29].

## Results

### PCNA and Mcm6 are tightly bound proteins

**Figure 1:**
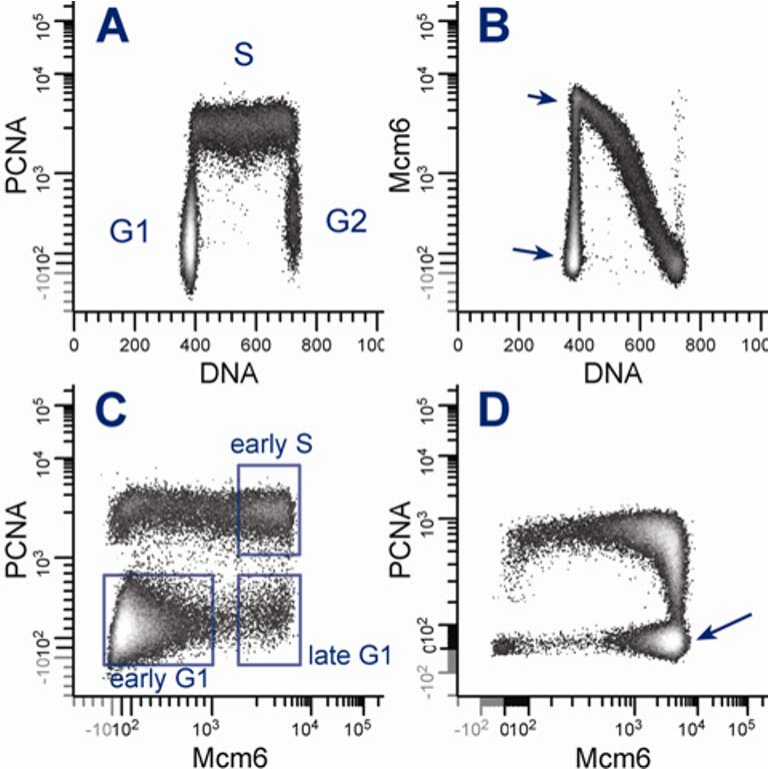
**Patterns of Mcm6 and PCNA expression**. Molt4 cells were permeabilized with Triton X-100, then fixed with MeOH prior to staining for Mcm6, PCNA, and DNA content. (**A**) G1 and G2/M clusters are defined by negative PCNA staining at 2C DNA and 4C DNA content, respectively. Positive PCNA staining delimits the S phase compartment. A few 4C G1 cells can be observed. (**B**) Mcm6 staining is bimodal in G1 phase (**arrows**), and the off-loading of Mcm proteins from chromatin during S phase is apparent. Note: the immunofluorescence axes are plotted on a hyperlog scale that produces a linear to log gradient and accommodates negative numbers and zero [43]. (**C**) a bivariate histogram of PCNA versus Mcm6 expression divides the G1/S interface into distinct clusters with very few cells classified as intermediate. (**D**) The pattern of Mcm6 expression in Molt4 cells varied with growth rate. In rapidly dividing cells, the high Mcm6 population was more pronounced (arrow) than in more slowly growing cells (Figure 1C, late G1). To simplify the bivariate patterns, G2/M cells were not included in C and D.

Discrimination between the Pre-RC components, bound PCNA and their respective soluble pools can be achieved by differential extraction with Triton X-100 [19,30]. This has been demonstrated with Mcm2, Mcm3 [17] and PCNA [31]. To confirm this, extend the observation, and to check the antibodies for non-specificity or cross-reactions, we examined Mcm2, Mcm6, PCNA, ORC3, and Cdc6 in whole cell lysates and those extracted with Triton X-100 before lysis. Western blots showed a 40 - 60% decrease in intensity in the detergent extracted lysates (Table 1; Additional file 1). A similar pattern was seen with cytometry (Additional file 2). Cyclins A2 and B1 are examples of proteins that appear completely extractable, whereas ORC2, ORC3, Cdc6, Mcm2, and Mcm6 all retain a measurable tightly bound component. Of the Pre-RC proteins that we examined by cytometry (ORC2, ORC3, Cdc6, Mcm2 and Mcm6), only Mcm2 and Mcm6 showed a bimodal distribution in G1 phase (Figure 1, Additional file 2).

**Table 1:** Fraction of remaining immuno-reactivity

Antigen	Immunoblot^a^	Cytometry^b^
Mcm6	0.6 ± 0.1 (3)	0.6
Cdc6	0.4 ± 0.0 (2)	0.3
PCNA	0.4 ± 0.1 (2)	0.2

^a ^Optical densities of bands from western blots of lysates from cells that were extracted with Triton X-100 prior to lysis were divided by the optical densities of bands from lysates. Means and standard deviations for multiple experiments (N) are listed.

^b ^Mean fluorescence intensities of Molt4 cells extracted with 0.5% Triton X-100 then fixed with methanol prior to staining were divided by the mean fluorescence intensities of Molt4 cells fixed in methanol then stained.

### At least 2 bound-Mcm states exist in G1

The data of Friedrich et al. [17] suggested that there were two distinct clusters in G1 with low and high immunoreactivity for tightly bound Mcm proteins. Figure 1 supports this idea. Figure 1A-C shows bivariate plots of DNA content versus PCNA (1A) for Molt4 cells with clearly delineated G1, S, and G2 clusters as expected from the work of Kurki et al. [19]. Figure 1B shows the expected pattern for Mcm proteins. There is a distinct low fluorescence cluster in G1 (long arrow) that is above the isotype control [17] and therefore positive for bound Mcm6. There is also a high positive cluster at the apex of the Mcm6 distribution and at the G1/S interface as defined by DNA content (short arrow). Figure 1C shows a bivariate plot for Mcm6 versus PCNA for the same cells using only the G1 and S phases (G2 cells were removed by a G2 gate on a DNA versus PCNA bivariate). This analysis clearly separates late G1 from early S phase. The cell population shown in Figure 1A-C was growing slowly at the time of fixation. Figure 1D shows a plot for Molt4 cells from a different experiment in which the cells were growing rapidly. The high Mcm6 cluster (arrow) is heavily populated in this case whereas the "early" G1 cluster is not. The simplest inference from these data is that the low Mcm6 expression G1 cluster represents an earlier time in G1 and the high Mcm6 expression G1 cluster represents a late time in G1.

### The low Mcm6 expression G1 cluster precedes the high expression G1 cluster

**Figure 2:**
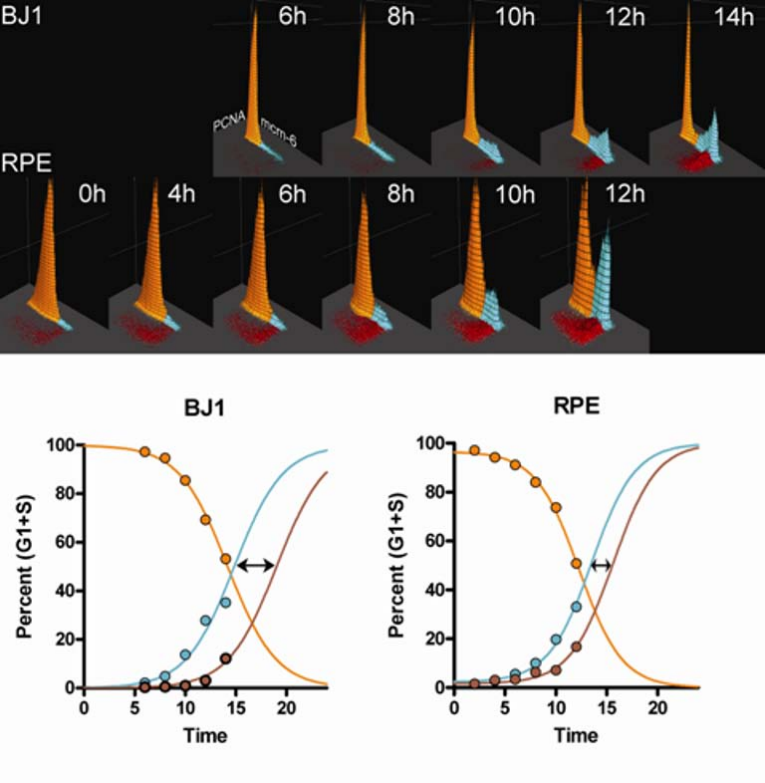
**Stimulation of serum starved cells**. BJ1 and RPE1 were serum starved for 24 h and then stimulated by addition of media with 10% serum. Samples were trypsinized, extracted with Triton X-100, fixed with MeOH, then stained and analyzed by flow cytometry. Data were preprocessed as in Additional file 3. **Top panel:**. the low Mcm6 G1 clusters (orange) decreased in frequency as a function of time, while the high Mcm6 (cyan) and PCNA positive S phase clusters (red-brown) increased. **Lower panel: **the fraction of cells residing in the low Mcm6 cluster (orange), high Mcm6 cluster, (cyan), and S phase (red-brown) were calculated as the percentage of G1 + S phase cells (Percent G1+S) and plotted as a function of time. The data were fit to constrained sigmoid equations with variable slopes (top = 100; bottom = 0). Kinetic analysis showed that the time to transit the high-Mcm6 state was 4.2 h for BJ1 and 2.1 h for RPE1 (arrows).

To formally test the previous statement, we serum starved BJ1 and RPE1 cells then stimulated them with media containing 10% serum. After 24 hours serum starvation, close to 100% of the cells are in the low Mcm6 expression G1 cluster (Figure 2, 0 h RPE1 cells). As early as 4 hours after serum stimulation, residence in the high expression G1 cluster has increased for RPE1. For BJ1, this can be seen as early as 8 h (Figure 2A). Kinetic analysis (Figure 2B) shows that the low expression G1 cluster decreases after serum stimulation as a function of time while the high expression G1 cluster increases, followed by an increase in S phase cells. For BJ1 cells, the Mcm6 levels in the low expression G1 cluster were distributed more narrowly with a lower center value, and therefore the identification of the boundary between the low and high clusters is more dramatic and easier to identify. However, the same pattern prevails in both cell lines. In experiments where the cells were sampled at later time points, from 20 to 28 h, the cells continued through S and G2/M phases in a semi-synchronous fashion. Note that the predicted time for 50% of the cells to enter the high cluster is 15 hours for BJ1 and 14 hours for RPE, and the time for 50% of the cells to enter S is 19 hours for BJ1 and 16 hours for RPE. Thus, at least under these conditions, residence in the high expression cluster is short compared to movement from the low expression cluster, as would be expected for uncommitted and committed G1 states.

### Presence of Mcm6 in the low expression G1 cluster

**Figure 3:**
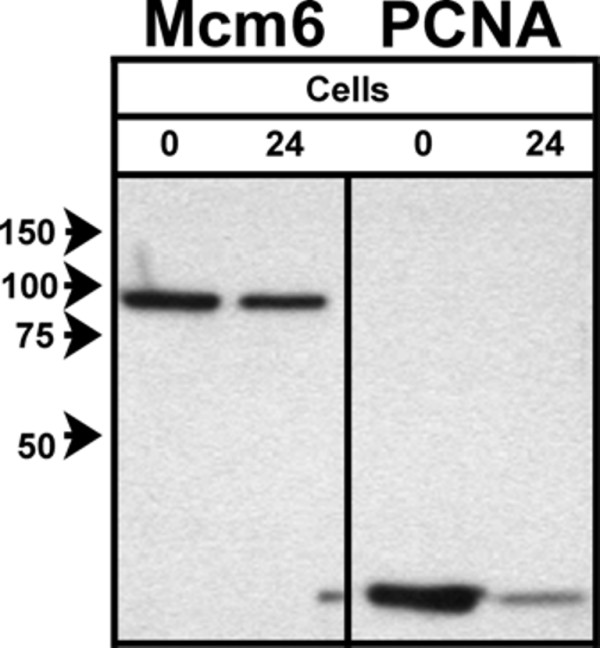
**Cells in the low Mcm6 cluster express loaded Mcm6**. BJ1 cells were harvested at 0 and 24 hours after serum starvation and prepared for electrophoresis after extracting the cells with Triton X-100 and washing. Equal cell numbers were loaded. The immunoblots were developed on X-Ray film that were then imaged and quantified. At 24 h, Mcm6 intensity was reduced by 25% and PCNA intensity by 72%. Under the conditions for serum starving BJ1 cells, there was an average of 5% of cells in S, G2 and M.

The presence of Mcm6 in the low expression G1 cluster is supported by Western blot of serum starved BJ1 cells. BJ1 cells were serum starved for 24 h before extraction with Triton X-100, washing, then lysate formation (Figure 3). Both Mcm6 and PCNA decreased in serum starved cells. The relatively strong level of Mcm6 compared to PCNA at 24 h of serum starvation indicates that the low Mcm6 population is not "essentially negative" for Mcm6 expression, but rather, distinctly positive. Therefore, we conclude that the low expression cluster of cells have loaded Mcm proteins. Different levels of quiescence can be achieved by serum starvation protocols. While the average time to S phase after stimulation may not be affected, the protein expression profile can be [32]. Our serum starvation experiments for BJ1 cells achieved an average of 95% of cells in G1. We did not growth arrest by confluence first. Therefore, our results do not say anything about true G0 cells and the expression of loaded Mcm proteins but do show that the low expression clusters in actively dividing cells are positive for loaded Mcm proteins.

### Serum starvation functionally separates the bound-Mcm states of G1

The restriction point (R) divides activated, uncommitted cells from those that are committed to the cell cycle. Cells that have not passed through "R" are not committed and arrest in G1 when serum or nutrients are withdrawn. Cells that are beyond R are committed to the cell cycle and behave like S, G2, and M cells - that is, when serum or nutrients are withdrawn, they complete the current cell cycle and the daughter cells arrest in the next G1. Here, we will refer to the uncommitted cells as G1a and the committed cells as G1p to distinguish them from G1pm and G1ps of Zetterberg [14] and various uses of G1A and G1B, since each nomenclature is defined by a measurement system.

**Figure 4:**
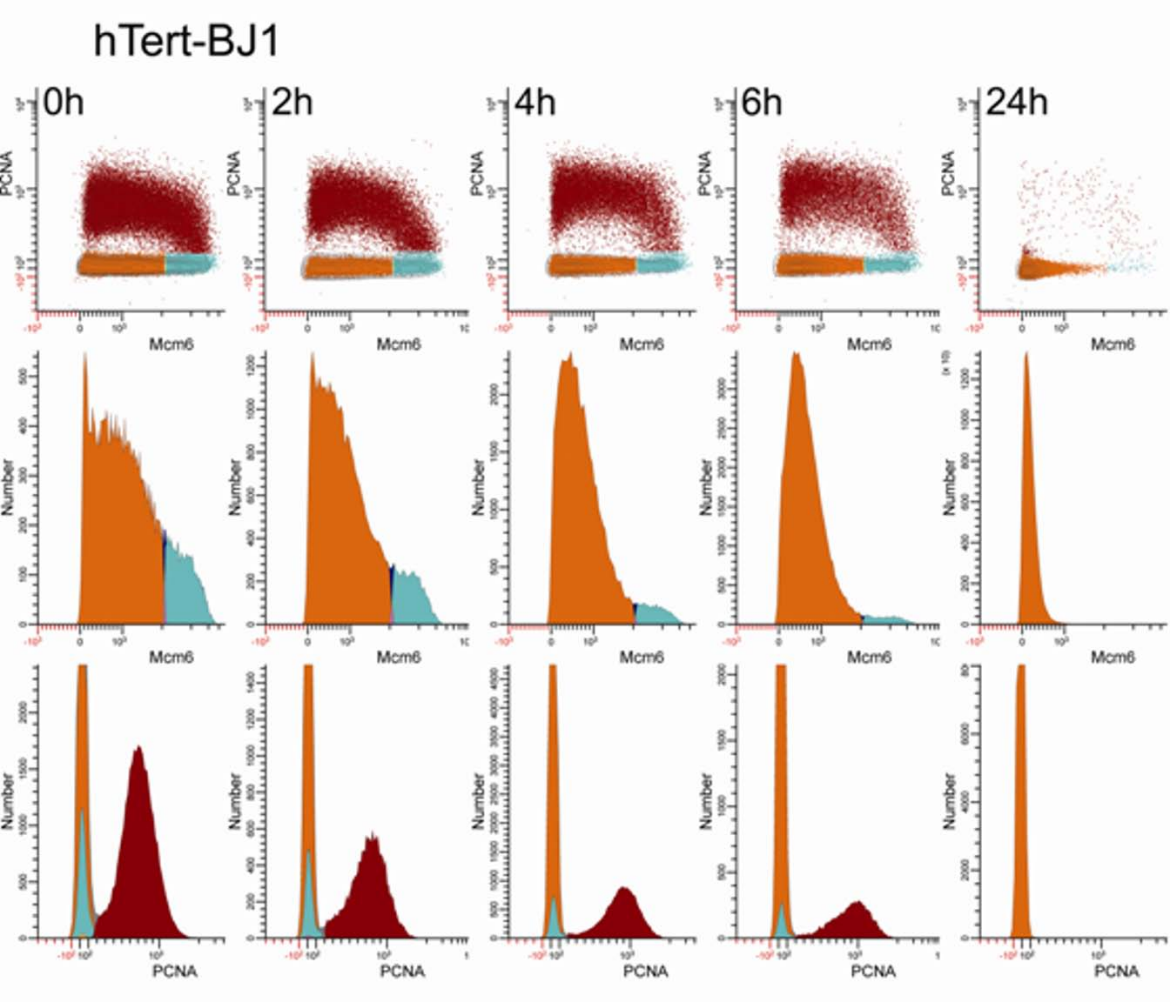
**hTert-BJ1 cells exit the high-Mcm6 G1 state with similar kinetics as S phase cells**. Exponentially growing BJ1 cells were switched to media containing 0.03% serum. At the indicated times, cells were harvested with trypsin and extracted with Triton X-100 prior to MeOH fixation, then stained, and subjected to cytometry. The regions for quantifying the fractions of cells in the three clusters were set as in Figure 2. Three views are shown. The first is a bivariate plot of G1 and S cells (top row). The second view, histograms of Mcm6 reactivity for G1 cells only, shows the decay of the high Mcm6 cluster and the decreasing intensity of the Mcm6 levels in the low Mcm compartment (middle row). The third view, histograms of PCNA reactivity for G1 and S cells, shows the decay of both the high Mcm6 cluster and the PCNA positive S phase cells (bottom row). Note: the Y axes in panels in the middle and bottom rows have variable scales.

To test whether the high Mcm6 G1 cluster was equivalent to G1p cells, we changed the growth media on actively dividing BJ1 and RPE1 cultures to media containing 0.03% FBS, and then tracked the changes in the low and high Mcm6 G1 clusters and the S phase fraction. Figure 4 shows G1 and S data for BJ1 cells. (Additional file 3 shows the preliminary processing and gates for cells that were eliminated from the analysis). The regions used to calculate the percentage of G1a (orange), G1p (cyan), and S (auburn) are color coded in two parameter plots of PCNA versus Mcm6 (top row). The middle row shows histograms for Mcm6 expression of G1 cells only, illustrating the decreasing frequency of G1p cells as a function of time. While there was clear evidence of a bimodal Mcm6 distribution in G1 at t = 0 h for BJ1 cells, there was no clear nadir. Therefore, the regions were set at the point where the slope of the frequency histogram first approaches zero on the right side of the dim population at t = 2 h. This setting was not sensitive to minor changes, and the behavior of each population was tested by setting a gap of varying width between the two regions and repeating the measures. As early as 2 h after serum withdrawal, the low Mcm6 G1 fraction began to increase and the population of high Mcm6 cells declined, suggesting that R is at the junction of these 2 populations. These changes continued as a function of time, and for BJ1 cells the process was complete by 24 h when 96% of the cells were resident in the low Mcm6 G1 cluster. The bottom row shows histograms for PCNA expression for both G1 and S cells, demonstrating the coordinated decrease in the fractions of G1p and S phase cells with time.

**Figure 5:**
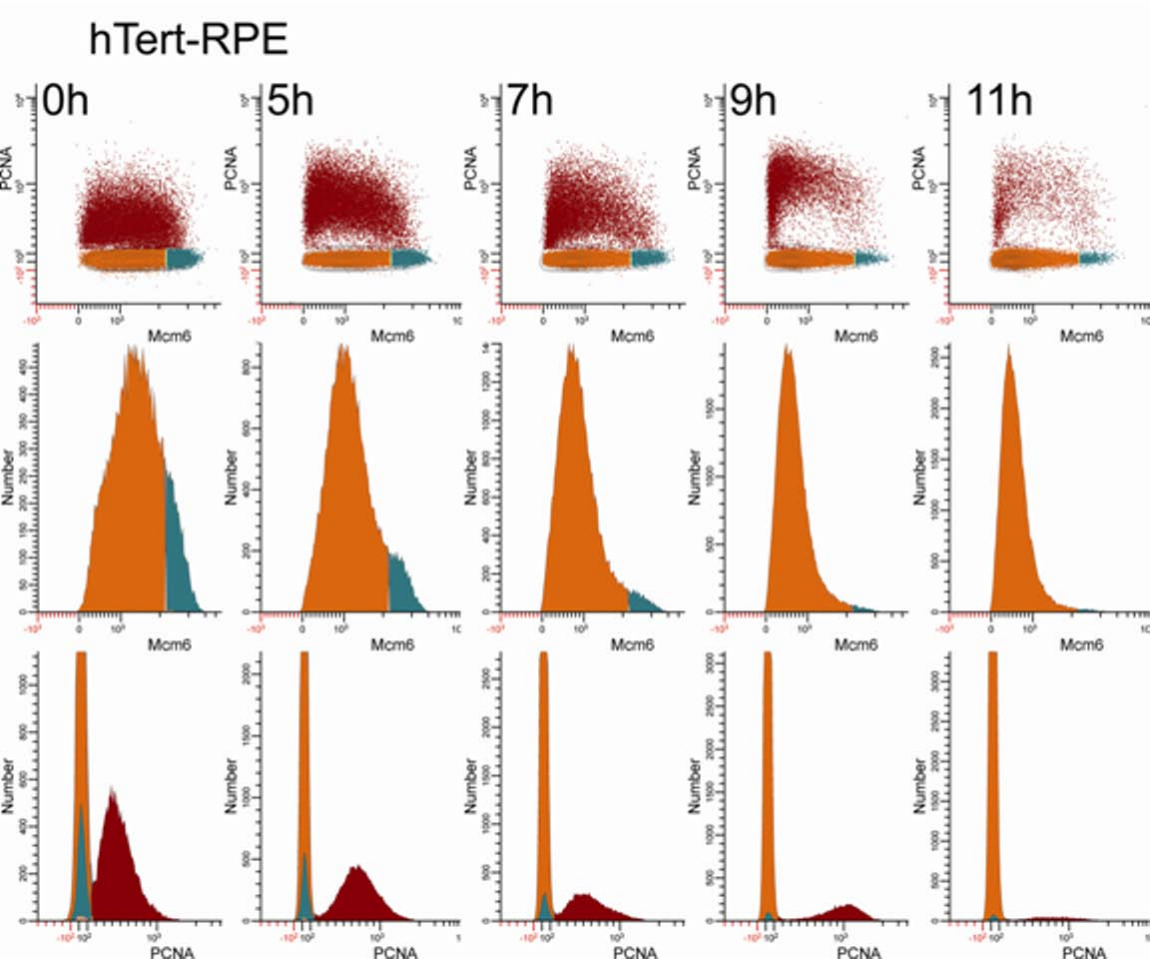
**hTert-RPE1 cells exit the high-Mcm6 G1 state with similar kinetics as S phase cells**. Exponentially growing RPE1 cells were switched to media containing 0.03% serum. At the indicated times, cells were harvested with trypsin and extracted with Triton X-100 prior to MeOH fixation, then stained, and subjected to cytometry. The regions for quantifying the fractions of cells in the three clusters were set as in Figure 2. The three views shown are the same as in Figure 4. The top row shows the quantitative relationship between measured parameters for G1 and S cells. The middle row shows the decay of the high Mcm6 cluster and the decreasing intensity of the Mcm6 levels in the low Mcm compartment. The third view shows histograms of PCNA reactivity for G1 and S cells and shows the decay of both the high Mcm6 cluster and the PCNA positive S phase cells (bottom row). Note: the Y axes in panels in the middle and bottom rows have variable scales.

Figure 5 shows a similar data presentation for RPE1 cells. This cell line displayed a different pattern as they entered serum-deprivation arrest. In rapidly dividing cells a large proportion of G1 cells are in the low Mcm6 cluster, but the mean fluorescence of this cluster is significantly higher than BJ1, suggesting that these cells assemble more of their replication complexes earlier than BJ1. The fraction of the high Mcm6 G1 cells was not easily discerned at t = 0 h, but by 5 h clear bimodality was evident (middle row). For RPE1, as for BJ1, the fraction of high Mcm6 G1 cells continuously decreased and the mean fluorescence of the low Mcm6 G1 population decreased (middle row) as did the S phase cells (bottom row).

**Figure 6:**
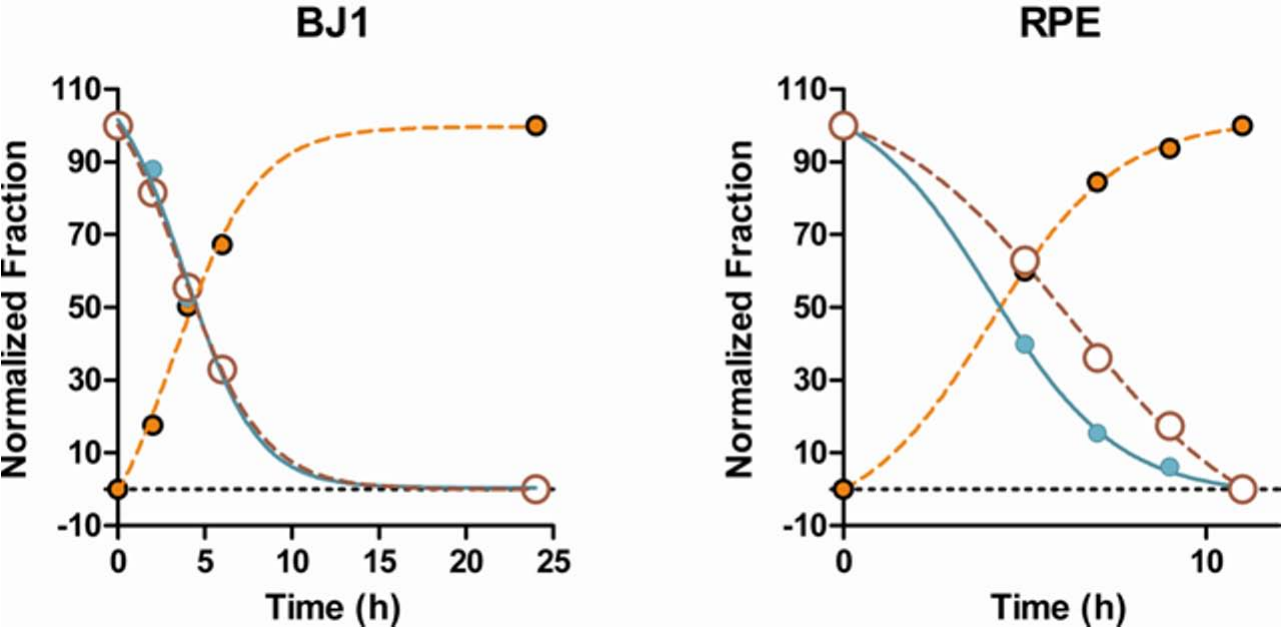
**BJ1 and RPE1 cells exit the high-Mcm6 G1 state with similar kinetics as S phase cells**. The frequencies of events in the low Mcm6 (orange), high Mcm6 (cyan), or S phase (brown-red) compartments, identified in Figures 4 and 5, were plotted versus time. The low Mcm-6 compartments increased in cell frequency, reaching ~95% at 24 h for both cell types. Note: the data have been normalized to 100 as the top value and zero for the bottom.

Figure 6 shows plots of the frequencies of G1a, G1p, and S phase cells for both cell lines (normalized to 100 for the top value for each type). In both cell, the high Mcm6 G1 fraction decreased at a rate that was equal to (BJ1) or faster than (RPE1) the S phase fraction. Therefore, we conclude that the high Mcm6 G1 cluster is equivalent to G1p cells. The transit times for G1p were 3.9 h for BJ1 and 4.1 h for RPE1, in good agreement with the results presented in Figure 2.

### Inhibitor studies

Because it is apparent that G1a cells lose Mcm6 fluorescence as a function of time in 0.03% serum, and presumably this equates to loss of pre-RC complexes, we needed to address the possibility that tentative G1p cells in both cell lines lost Mcm6 expression - i.e., moved backwards into the low Mcm6 cluster, rather than having progressed into S. To address this, we used cell cycle inhibitors.

**Figure 7:**
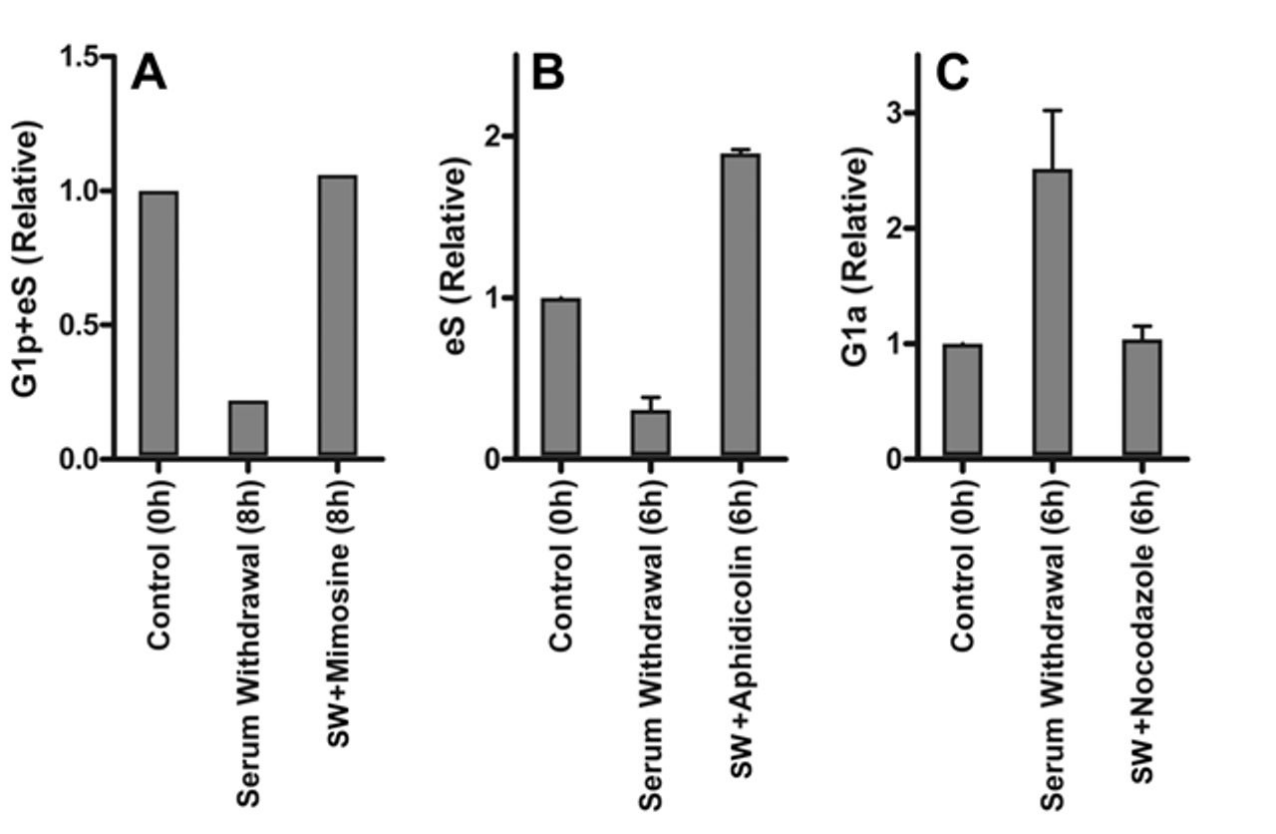
**Effect of cell cycle inhibitors**. Exponentially growing BJ1 cells were switched to medium with 0.03% FBS and inhibitors. Controls (no switch or addition) and treated cells were extracted with TX-100, fixed with MeOH. Controls were processed at time zero; treated samples were processed at 6 or 8 hours. Samples were then stained for Mcm6, PCNA, phospho-S10-histone H3, and DNA and subjected to cytometry as described in the Methods section. The percentages of G1p+eS, eS, G1a were normalized to the same fraction in the control sample. Normalized eS (early S phase), G1p+eS (high-Mcm6 cells+ early S phase), and G1a (low-Mcm6 G1 cells) are plotted for cells treated with mimosine (**A**), aphidicolin (**B**), and nocodazole (**C**). Serum withdrawal stopped forward movement of cells from the G1a compartment but not G1p or S (middle bars). Serum withdrawal plus mimosine prevented cells from entering or exiting the G1p compartment (**A**). Serum withdrawal plus aphidicolin arrested cells in S (**B**). The frequency of eS increased over 6 hours as cells moved from G1p to eS. Serum withdrawal plus nocodazole prevented cells from entering the G1a compartment (**C**).

Mimosine blocks the cell cycle at late G1 and S if added to asynchronous cultures [33]. Mimosine at 120 μg/ml (600 μM final) was added to BJ1 cells at the time of serum withdrawal and cells were harvested after 8 h. PCNA immunofluorescence was significantly reduced in the presence of mimosine (Additional file 4). Because of this we could not readily distinguish the high Mcm6 G1 cluster from early S phase, and therefore used Mcm6 versus DNA plots to quantify G1p + early S (eS) cells. Analysis presented in Figure 7A shows that in the absence of serum the frequency of G1p+eS cells decreased in BJ1 cultures but in the presence of mimosine it increased slightly relative to control cells in 10% serum at t = 0 h. This is consistent with a late G1 and S phase block. We conclude that high Mcm6 G1 cluster BJ1 cells did not significantly progress into S but did not move backwards to the low Mcm6 G1 cluster either.

Aphidicolin inhibits DNA elongation but not initiation of DNA primer formation by inhibiting DNA polymerase δ/ε [34], therefore, it blocks the progression of cells beyond early S phase and any cells already in S phase are immobilized. At the same time serum was removed from cycling cultures of BJ1 cells, aphidicolin at 1 μg/ml was added. The results are shown in Figure 7B. Cells in the early S compartment (high Mcm6 and PCNA) decreased during serum starvation but increased during serum starvation in the presence of aphidicolin. Essentially the same results were determined with RPE1 cells (not shown). This indicates that cells within the high Mcm6 G1 cluster moved into early S rather than to the low Mcm6 state.

**Nocodazole **inhibits polymerization of tubulin and thus prevents formation of the mitotic spindle by inhibiting microtubule elongation. When nocodazole at 50 ng/ml was added to BJ1 cells at the start of serum withdrawal, the low Mcm6 cluster (G1a) did not change (Figure 7C). Cells were prevented from entering G1a by the nocodazole block in M, and they were prevented from leaving by serum withdrawal.

The mimosine, aphidicolin, and nocodazole data confirm the uni-directional flow of cells through G1 phase. When serum is removed, the high Mcm6 cluster G1 cells don't return to a low Mcm6 state, but rather continue on and accumulate in early S in the presence of aphidicolin or mimosine. Cells only enter the low Mcm6 G1 state from mitosis and this entry is blocked in the presence of nocodazole. Therefore, the low Mcm6 state in G1 is equivalent to G1a and the high Mcm6 state in G1 is equivalent to G1p.

## Discussion

Earlier results from Friedrich et al. suggested that in CV-1 cells G17 might be subdivided by the expression of low and high levels of chromatin loaded Mcm proteins [17]. To ask whether this subdivision demarked uncommitted and committed G1, we developed a cytometric assay that included DAPI to stain DNA and antibodies reactive with Mcm6, PCNA, phospho-S10-histone H3. PCNA was included to cleanly separate S phase from late G1. It was previously known that G2 cells would have minimal levels of bound Mcm proteins but loading of pre-RCs begins in late mitosis, and therefore, we included a mitotic marker. This 4 color assay clearly showed in three human cell lines (Molt4, BJ1, RPE) that in actively replicating cells, G1 is subdivided into at least two states characterized by low and high levels of loaded Mcm6 (and by inference, the Mcm complex). We showed that the low level expression state is variable with respect to the average or modal levels that are expressed. We suspect that this variability is related to the rate of G1 transit (e.g., Figure 1), and we suspect that the high levels are relatively constant, but neither of these ideas have been tested rigorously. Further, we showed by cytometric analysis of serum stimulation experiments, serum withdrawal experiments, and stathmokinetic experiments that the low level state is correlated with the uncommitted G1 state and that cell cycle commitment is correlated with the high level state. Finally, we observed the lowest expression of bound Mcm6 in mitotic cells (see Additional files 4, 5, and 6). We did not see any evidence of bimodality in mitotic cells, and therefore the pre-RCs that are bound in mitosis are below the level of detection in this assay.

For this study, we have treated G1 as composed of two states defined by levels Mcm levels. However, during this study we observed distributions of Mcm6 in G1 with more structure than this simplified view. At a minimum, we could model G1 as composed of a basal population equal to the mitotic cells with the lowest levels of bound Mcm proteins, a center population with a variable modal level of bound Mcm proteins, and finally a high population with maximum levels of bound Mcm proteins. The mitotic lowest population should set the biological bottom; the variable center population should vary with growth conditions, and the maximum population should define the levels necessary to enter S phase (e.g., see Additional files 5 and 6). Our results concur with Friedrich et al. in that the level of Mcm proteins detected in the high Mcm cluster appear to be equivalent to the highest levels achieved in cell populations fixed and stained for both bound and unbound Mcm proteins. This may mean that at equilibrium, at the G1 → S transition, all the Mcm proteins that can be bound are bound. If that speculation proves to be true, then it would be interesting to know whether the max levels of Mcm proteins fluctuate as a function of growth rate as well as the center population. Support for any of these speculative remarks will require significant further experimentation.

Mukherjee at al. [18] have correlated high levels of bound Mcm proteins, cdc45, hyper-phosphorylated Rb, and cyclin E expression with the committed period of G1. They showed that synchronized cell populations (starve and release) treated with RNA polymerase II inhibitors were not arrested during a period when most cells would be in late G1, and therefore, these parameters were correlated with cell cycle commitment. These results are in agreement with the findings of Chuang et al. for stimulated quiescent human epithelial cells that assign Cdc6 binding, Mcm loading, and Cdc7 expression to late G1, dependent on cyclin E activity [23]. Our data are in agreement with both of these studies, but further illustrate that even in actively cycling cells there is a measureable period in late G1 in which Mcm proteins are maximally bound to chromatin. Further, our results strongly suggest that this period is universally passed through prior to PCNA loading and the start of S phase in human somatic cells. Our data do not address the conclusion from both papers that loading occurs solely in late G1. We have focused on actively dividing asynchronous cells - an area that cytometry addresses well. We have presented a starve and stimulate experiment in Figure 2 for hTert immortalized human fibroblasts and epithelial cells. As stated in the text associated with Figure 3, our starvation experiments were performed to determine the sequence of Mcm loading from low to high, and it is likely that we have not worked with cells as deeply quiescent as either Mukerjee et al. or Chuang et al.

The low to high sequence of Mcm expression in G1 may seem obvious from primary asynchronous data as shown by Friedrich et al [17] and more explicitly in this paper (early S phase associates with the high Mcm G1 cluster and late S through mitosis associates with the low Mcm G1 cluster (Figure 1, Additional files 4, 5, 6)). Nevertheless, it was necessary to test the hypothesis. Single static samples of the type presented in Figure 1 could arise from a more complex expression program than "basal → max → basal" and that complexity would become obvious in experiments like that in Figure 2.

Any conclusion about ORI loading rests on the assumption that all or most of the bound Mcm proteins are associated with ORI. In mammalian cells, origins of replication are not well defined (e.g., [10] are listed by Schepers and Papior for human cells [35]), and therefore comprehensive temporal licensing studies have not been done. More Mcm proteins are bound relative to the number of bound ORC complexes and most Mcm proteins do not co-localize with replication forks or with ORC [see references in [36]]. There is some evidence that much of the excess Mcm loading is related to dormant ORI that respond to replication fork stalling [36,37]. In contrast to higher eukaryotes, significant progress has been made in yeast toward identifying all origins (ARS in *S. cerevesiae*) using genome-wide information and technologies [35,38]. In *S. pombe*, which has defined and known origins without consensus sequences, there is a distribution of the efficiency with which origins are used during S phase [see references in [39]]. High frequency origins are used often (e.g., once every two cycles) and lower efficiency origins are used less (e.g., one in ten cycles) [reviewed in [35]]. There also appears to be a hierarchal order of ORC binding beginning in mitosis according to origin efficiency that appears at least partly dependent on affinity [39]. In *S. pombe*, evidence suggests that high efficiency origins are bound early and low efficiency origins are bound late and that this order is followed by Mcm loading and subsequently Cdc45 loading [39]. Therefore, at least in actively dividing somatic cells, the possibilities are that low efficiency and/or dormant origins are licensed in the G1p state (maximum loaded Mcm2-7). Alternatives are that no particular origins are licensed during this period or more complex scenarios coupled with transcription promoter sites could be entertained. It seems like genome-wide efforts will be required to determine whether these ideas have any merit. Since cells resident in this cytometrically defined, high Mcm G1 state could be sorted, perhaps FACS coupled with genome-wide technologies [e.g., [40]] could provide comprehensive answers.

It is clear from our data that Mcm binding occurs throughout G1 in both quiescence-stimulation experiments and in actively cycling populations. It is probable, that in our serum starvation experiments we did not achieve "deep" quiescence (see section: Presence of Mcm6 in the low expression G1 cluster) and that in true G0 cells (e.g., fibroblasts that have been arrested by both density and starvation), most of the binding occurs during late G1. Even in our experiments (see BJ1 cells at 14 h in Figure 2), the Mcm levels of the G1p cells is ~10 fold greater than that of the G1a cells, which would lead one to conclude that most of the loading occurs in late G1 during cell cycle re-entry, whereas it is less clear in actively dividing cells. The Mcm levels are ~4 fold higher in G1p BJ1 than G1a (Figure 2A 0 h), and ~2 fold higher in RPE G1p compared to G1a (Figure 2B 0 h). However in Molt4 the levels of the two states are distinctly different with almost all of the loading occurring in G1p whether or not the population is growing slow or fast. These are also lymphoma cells, and therefore, deregulation of G1 progression is highly probable. However, in other analyses (actively dividing iHUVEC), we have observed clear evidence of non-linear loading in G1, i.e., a large fraction of G1a cells separated from a large fraction of G1p cells as we observed for Molt4 cells (e.g., Figure 1C an 1D). This can be thought of as a switch from a low loading rate to a high loading rate at the time of switching from uncommitted to committed G1.

## Conclusions

Overall, our data support the idea that mitotic cells represent the lowest bound Mcm levels in actively cycling cells; that the rate of chromatin loading in uncommitted G1 cells is variable, and that committed G1 cells are characterized by a maximum level achieved just prior to S. The modal level of binding in an asynchronous, actively cycling population appears to be higher in fast replicating populations and lower in slower replicating populations. This supports the idea that the differences in rates of loading between cycling cells and stimulated G0 cells is a continuum and dependent on the rates of synthesis and activation of cyclin E and Cdk2 for re-entry and perhaps D cyclins and Cdk4/6 (E2F activity in both cases) as suggested by Chuang et al. [23].

## Methods

### Cell lines, culture and fixation

Molt4 (human T cell lymphoma cell line) was obtained from ATCC (CRL-1582); hTERT-BJ1 (BJ1) and hTERT-RPE-1 (RPE) were from Clontech, Mountain View, CA. Molt 4 cells were grown in RPMI Medium 1640, BJ1 cells were grown in 80% Dulbecco's modified Eagle medium (DMEM) and 20% Medium 199 supplemented with 4 mM L-glutamine and 1 mM sodium pyruvate. RPE1cells were grown in D/MEMF-12 containing 2 mM L-glutamine and 0.375% sodium bicarbonate. These media routinely contained 10% fetal bovine serum (FBS) (Cambrex, Charles City, IA) and 50 μg/ml gentamicin sulfate (Fisher, Pittsburgh, PA). Media and supplements were from Gibco, Carlsbad, CA. Cells were grown at 37°C in a humidified atmosphere with 5% CO_2_.

Cells were serum starved by washing the cells in PBS and then adding the appropriate medium with 0.03% FBS. In some experiments aphidicolin (Alexis, San Diego, CA), mimosine (Sigma, St. Louis, MO), or nocodazole (Sigma) were added at the time of serum withdrawal.

Adherent cells were harvested with trypsin and cell counts were taken on a Coulter Counter (Coulter Electronics, Hialeah, FL). Washed cells were either fixed directly in 90% methanol at -20°C or first detergent extracted (0.5% Triton X-100, 0.2 μg/ml EDTA, 1% BSA in PBS [30] for 10 min at 4°C before methanol fixation. Detergent solution was added at 40 μl/10^6 ^cells. Final cell concentration was 2.5 × 10^6 ^cells/ml.

### Antibodies

The following antibodies were used for cytometry and immunoblotting: Phycoerythrin-conjugated and unconjugated anti-Mcm6 (PE-Mcm6), clone K1-Mcm6, (BD Biosciences); fluorescein isothiocyanate-conjugated and unconjugated anti-PCNA (FITC-PCNA), clone PC10 (BD Biosciences); BM28 (anti-Mcm2), clone 46, (BD Biosciences); anti-Cdc6, clone DCS-180, (Upstate, Lake Placid, NY); anti-ORC3, clone 1D6, (Upstate); Alexa Fluor 647-conjugated anti-phospho-S10-histone H3 (A647-pHH3) (Cell Signaling, Danvers, MA); FITC-conjugated goat anti-mouse F(ab)^2 ^(BD Biosciences).

### Flow cytometry

Fixed cells were washed and immunostained with primary antibody for 90 min at 4C in PBS with 2% BSA. If the primary antibody was unconjugated, cells were washed twice and immunostained with the appropriate conjugated secondary antibody. DNA was stained with DAPI at 0.25 - 1 μg/10^6 ^cells.

Cells were measured with a BD LSR II flow cytometer (BD Biosciences, San Jose, CA). Cells were excited with the 355 nn, 488 nm, and 633 nm lasers. Final filters were 450/50, 530/30, 575/26 and 670/20 nm. Data were analyzed with WinList 3D 6.0 (Verity Software House, Topsham, ME).

### Laser scanning cytometry

Cells were grown on 35 mm glass bottom microwell dishes (MatTek, Ashland, MA) that had been treated with FBS for 1 h to coat the glass with fibronectin. After culture, cells were extracted with detergent, fixed in methanol and immunostained as above. Plates were scanned with a Compucyte (Westwood, MA) iCyte using a 405, 488, and 633 nm excitation and 465/40, 530/30, 575/20 and 675/50 nm emission for DAPI, FITC, PE, and A647 fluorescence, respectively.

### Electrophoresis and western blotting

Known numbers of cells were washed and lysed directly in 5% SDS lysis buffer (0.137 M NaCl, 2% Nonidet P-40, 5% SDS, 1% sodium deoxycholate, 20 mM Tris, pH 8.0, 2 mM PMSF, 10 μl/ml protease inhibitor cocktail (P8340, Sigma)) or first extracted with detergent as described above, washed twice in 1 ml PBS at 4°C with centrifugation for 1 min at low speed in a swinging bucket microfuge (Fisher, Model 59A) before lysis buffer was added to the pellet.

Equal cell numbers or equal amounts of protein were loaded on 10% polyacrylamide discontinuous mini-gels (Bio-Rad, Hercules, CA) and electrophoresed conventionally [41]. Gels were electrophoretically blotted [42] onto Immobilon-P membrane (Millipore, Bedford, MA) without methanol for 15 min at 100 V. Antigens were visualized by immunostaining with the appropriate antibody and alkaline phosphatase conjugated secondary antibody (Promega, Madison, WI) using chemiluminescent detection with CDP-Star substrate (Tropix, Bedford, MA). Blots were exposed to X-ray film, developed, then imaged with a BioRad GelDoc EQ. Quantification was performed using Quantity One 4.1.1 software (Bio-Rad).

## Abbreviations

A488: Alexa Fluor 488; A647: Alexa Fluor 647; APC/C: anaphase promoting complex/cyclosome; BSA: bovine serum albumin; BJ1: hTert-BJ1; Cdk: cyclin dependent kinase; DAPI: 4',6-diamidino-2-phenylindole; EDTA: ethylenediaminetetraacetic acid; FACS: fluorescence activated cell sorting; FITC: fluorescein isothiocyanate; Mcm: mini-chromosome maintenance; Mcm2-7: Mcm protein complex; ORC: origin recognition complex; ORI: origins of replication; PCNA: proliferating cell nuclear antigen; PHH3: phospho-S10-histone H3; PMSF: phenylmethanesulfonyl fluoride; pre-RC: pre-replication complex; RPE: hTert-RPE-1; R: restriction point.

## Competing interests

The authors declare that they have no competing interests.

## Authors' contributions

PSF conceived, designed, and executed experiments; analyzed data, and drafted/edited the manuscript. JWJ conceived the study and helped design experiments; analyzed data, and edited/co-wrote the manuscript. Both authors have read and have approved the final manuscript.

## Supplementary Material

Additional file 1 **Western blot detection of tightly bound proteins**. BJ1 cells were extracted with 0.5% Triton X-100 before solubilization for electrophoresis. 10^5 ^cells were loaded in each lane. Western blots were probed with antibodies to the indicated proteins as described in Methods. (A) the two antibodies used in the study. (B) antibodies to two other replication complex proteins and cyclin B1, which does not appear to be tightly bound at a detectable level in asynchronously growing cells. In addition to detection of tightly bound antigen, these blots also demonstrate the high specificity of the Mcm6 and PCNA antibodies, since cross reacting bands are not evident. Click here for file

Additional file 2 **Cytometric detection of tightly bound proteins**. Exponentially growing Molt4 cells were directly fixed in methanol (MeOH) or first treated with Triton X-100, fixed with MeOH (TX-100/MeOH); then stained for Cdc6, ORC2, ORC3, Mcm2, cyclin A2, using indirect staining with FITC conjugated goat anti-mouse antibodies and counter-stained for DNA content (DAPI), then measured by cytometry. In the first two panels (Cdc6), a secondary antibody control was run (blue outlines), demonstrating elevated background in MeOH-fixed cells as expected. Secondary only controls were not run with the remaining antigens. In all cases, a significant loss of signal was achieved after detergent extraction. The results agree quantitatively with Western blots for Cdc6 (Table 1) and agree qualitatively for the other antigens (Additional file 1). The detergent extracted pattern for Mcm2 demonstrates bimodality in G1 (arrows), declining signal in S phase, and a return to baseline in G2+M. This pattern is equivalent to that for Mcm6. Click here for file

Additional file 3 **Cytometry data preprocessing for figures **2**and **4. Cytometry data for RPE1, stained for PCNA (FITC), Mcm-6 (PE), phospho-S10-histone H3 (A647), and DNA (DAPI) are shown. (A) aggregate and debris discrimination: singlet cells were included in region R1 based on integrated (UV-440-A) versus peak (UV-440-H) DAPI signal. All subsequent data were gated on R1. (B) Mitotic discrimination: mitotic cells were included in region R2 based on elevated histone H3 phosphorylation. All subsequent data were Boolean "NOT" gated on R2. (C) G2 discrimination: G2 and 4C G1 cells were included in region R3 based on 4C DNA content and absence of bound PCNA expression. All subsequent data except (D) were NOT gated on R3. (D) 4C G1 cell discrimination: 4C G1 cells (Mcm6 positive) and negative 4C cells were included in region R4. All subsequent data were NOT gated for R4. This is ~redundant with R3. (E) abnormal large and small cell discrimination: abnormally small events were included in R5. Large cells with low Mcm6 levels were identified as abnormal large G1 cells (i.e., based on size, they should have been Mcm6-high). Subsequent data were NOT gated for R5 and R6. (F) resultant plot after sequential Boolean logic (R1 NOT (R2 OR R3 OR R4 OR R5 OR R6)) applied to data. G1 (orange and cyan) and S phase (red-brown) data result. Data were also compensated conventionally for spectral overlap between FITC and PE (not shown). Removal of cells in R6 is conservative in a cell cycle sense. Their size suggests that they should have entered S phase, since they are larger than the average cell in early S. We see these in variable numbers in all three hTert immortalized lines with which we have worked. Since they express Mcm6 very low, they may be out-of-cycle for unknown reasons. If these cells are included, the information in Figures 2 and 4 - 6 do not change, suggesting that they represent an offset in the G1a compartment. Click here for file

Additional file 4 **Mimosine treatment reduces the level of bound PCNA from S phase cells**. BJ1 cells were grown in 10% or 0.03% serum in the presence and absence of 120 μg/ml mimosine. After 8 h, treated cells were extracted with Triton X-100 and fixed in methanol (see Methods) before staining for cytometry. Control cells were harvested at time zero. Red dots = mitotic cells. SS = serum starvation. Click here for file

Additional file 5 **Laser scanning cytometry**. Exponentially growing BJ1 were fixed and stained for Mcm6, PCNA, phospho-S10-histone H3, and DNA as described in Methods. The volume of the staining reaction is greater than flow cytometry samples, but is otherwise the same. These data verify that both signals are nuclear (green = PCNA and orange = Mcm6). Mitotic cells were gated (not shown) and color-coded magenta. The pixels representing the rare mitotic events were made larger in Adobe Photoshop so that they would stand out. These data also support the notion that a more sophisticated analysis of bound Mcm6 versus PCNA might segment G1 into three, rule-based states, G1a_1 _[G1a cells with the lowest expression of Mcm6 and PCNA, equivalent to mitotic cells] (B); G1a_2 _[G1a cells (green) expressing Mcm6 at significant but sub-maximal levels] (C), and G1p cells (cyan), defined as the Mcm6 level at which cells enter S (D). Late S phase cells (red) are shown for visual comparison (E). Click here for file

Additional file 6 **Flow cytometry of Mcm-6 expressed G1 cells**. RPE1 cells were grown exponentially (A) or serum starved for 25 h (B). The cells were fixed then stained for Mcm6, PCNA, phospho-S10-histone H3, and DNA. Mitotic cells (blue histogram) were gated on DNA and phospho-S10-histone H3; early S phase cells (red-brown histogram) were gated on DNA and PCNA, and G1 cells (cyan histogram) were gated on DNA and (absence of) PCNA. The Mcm6 frequency distributions from each gate were overlayed to show the relative expression of each. The distributions are plotted with Y scaling set to the frequency peak height, therefore frequencies are normalized (labeled "number"). The exponentially cycling cells are larger and have higher background fluorescence and therefore, broader coefficients of variation (note the broader mitotic and early S distributions). The key information is that the mitotic levels and S phase levels set the bounds of the distribution (min and max) and that the transition state between them is variably populated with slower growing populations shifted to the left and faster growing populations shifted to the right. Click here for file
