# Supplementary figures and images for: Cytometry of chromatin bound Mcm6 and PCNA identifies two states in G1 that are separated functionally by the G1 restriction point1
Source: BMC Cell Biol. 2010 Apr 16;11:26. doi: 10.1186/1471-2121-11-26 (PMC2882901; doi:10.1186/1471-2121-11-26)

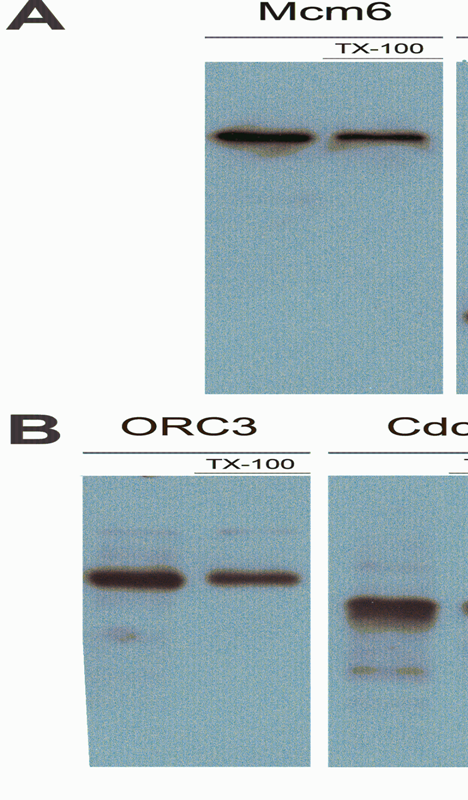

Supplement: Additional file 1 — Western blot detection of tightly bound proteins. BJ1 cells were extracted with 0.5% Triton X-100 before solubilization for electrophoresis. 105 cells were loaded in each lane. Western blots were probed with antibodies to the indicated proteins as described in Methods. (A) the two antibodies used in the study. (B) antibodies to two other replication complex proteins and cyclin B1, which does not appear to be tightly bound at a detectable level in asynchronously growing cells. In addition to detection of tightly bound antigen, these blots also demonstrate the high specificity of the Mcm6 and PCNA antibodies, since cross reacting bands are not evident. [file 1471-2121-11-26-S1.TIFF]

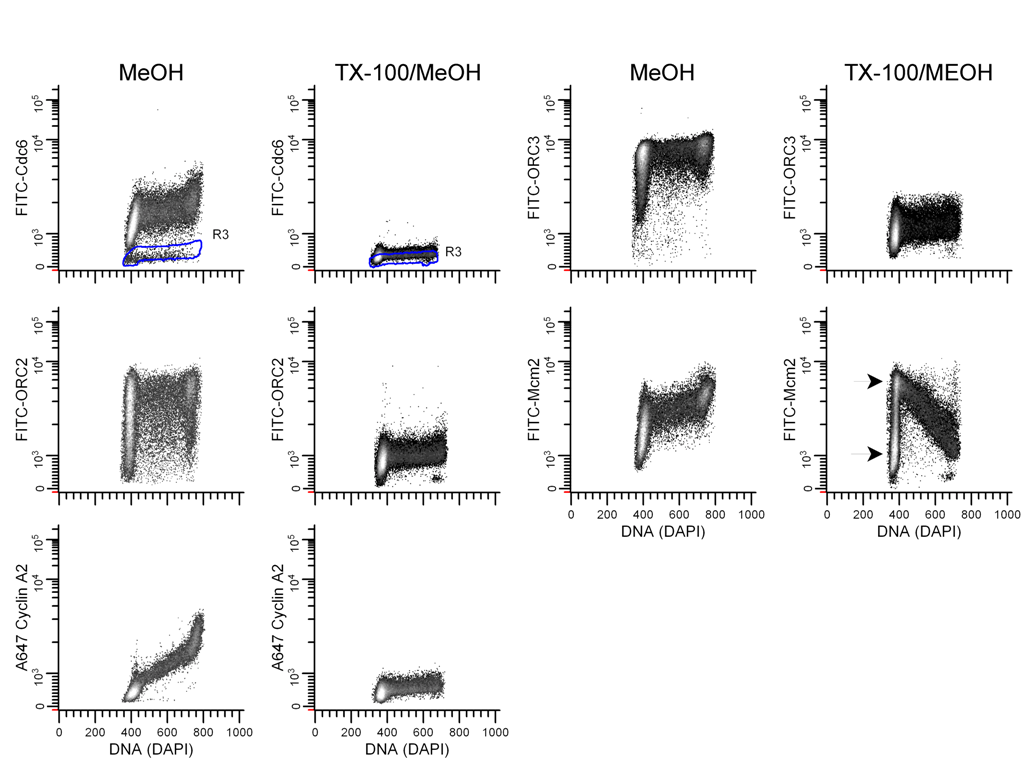

Supplement: Additional file 2 — Cytometric detection of tightly bound proteins. Exponentially growing Molt4 cells were directly fixed in methanol (MeOH) or first treated with Triton X-100, fixed with MeOH (TX-100/MeOH); then stained for Cdc6, ORC2, ORC3, Mcm2, cyclin A2, using indirect staining with FITC conjugated goat anti-mouse antibodies and counter-stained for DNA content (DAPI), then measured by cytometry. In the first two panels (Cdc6), a secondary antibody control was run (blue outlines), demonstrating elevated background in MeOH-fixed cells as expected. Secondary only controls were not run with the remaining antigens. In all cases, a significant loss of signal was achieved after detergent extraction. The results agree quantitatively with Western blots for Cdc6 (Table 1) and agree qualitatively for the other antigens (Additional file 1). The detergent extracted pattern for Mcm2 demonstrates bimodality in G1 (arrows), declining signal in S phase, and a return to baseline in G2+M. This pattern is equivalent to that for Mcm6. [file 1471-2121-11-26-S2.TIFF]

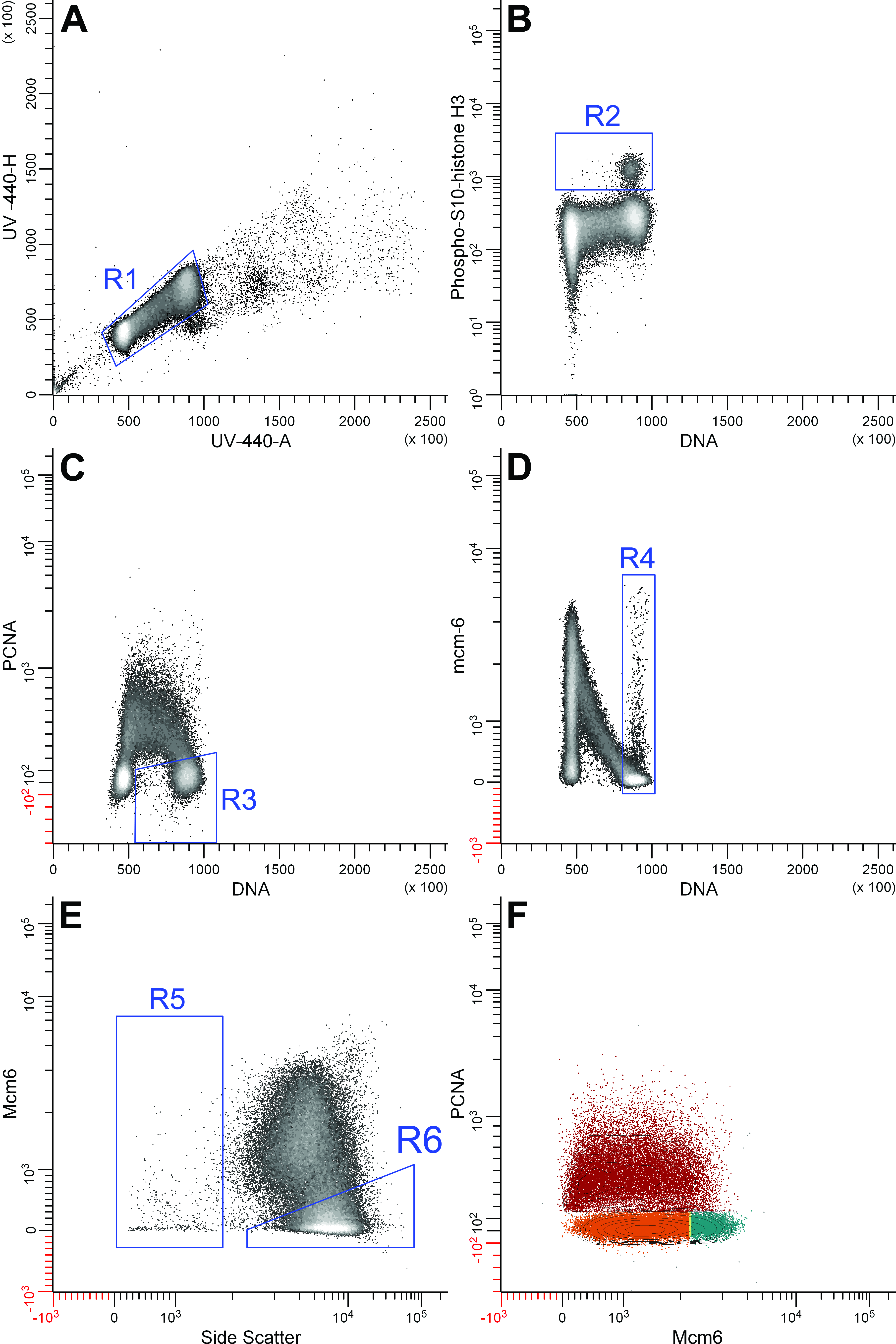

Supplement: Additional file 3 — Cytometry data preprocessing for figures 2and 4. Cytometry data for RPE1, stained for PCNA (FITC), Mcm-6 (PE), phospho-S10-histone H3 (A647), and DNA (DAPI) are shown. (A) aggregate and debris discrimination: singlet cells were included in region R1 based on integrated (UV-440-A) versus peak (UV-440-H) DAPI signal. All subsequent data were gated on R1. (B) Mitotic discrimination: mitotic cells were included in region R2 based on elevated histone H3 phosphorylation. All subsequent data were Boolean "NOT" gated on R2. (C) G2 discrimination: G2 and 4C G1 cells were included in region R3 based on 4C DNA content and absence of bound PCNA expression. All subsequent data except (D) were NOT gated on R3. (D) 4C G1 cell discrimination: 4C G1 cells (Mcm6 positive) and negative 4C cells were included in region R4. All subsequent data were NOT gated for R4. This is ~redundant with R3. (E) abnormal large and small cell discrimination: abnormally small events were included in R5. Large cells with low Mcm6 levels were identified as abnormal large G1 cells (i.e., based on size, they should have been Mcm6-high). Subsequent data were NOT gated for R5 and R6. (F) resultant plot after sequential Boolean logic (R1 NOT (R2 OR R3 OR R4 OR R5 OR R6)) applied to data. G1 (orange and cyan) and S phase (red-brown) data result. Data were also compensated conventionally for spectral overlap between FITC and PE (not shown). Removal of cells in R6 is conservative in a cell cycle sense. Their size suggests that they should have entered S phase, since they are larger than the average cell in early S. We see these in variable numbers in all three hTert immortalized lines with which we have worked. Since they express Mcm6 very low, they may be out-of-cycle for unknown reasons. If these cells are included, the information in Figures 2 and 4 - 6 do not change, suggesting that they represent an offset in the G1a compartment. [file 1471-2121-11-26-S3.TIFF]

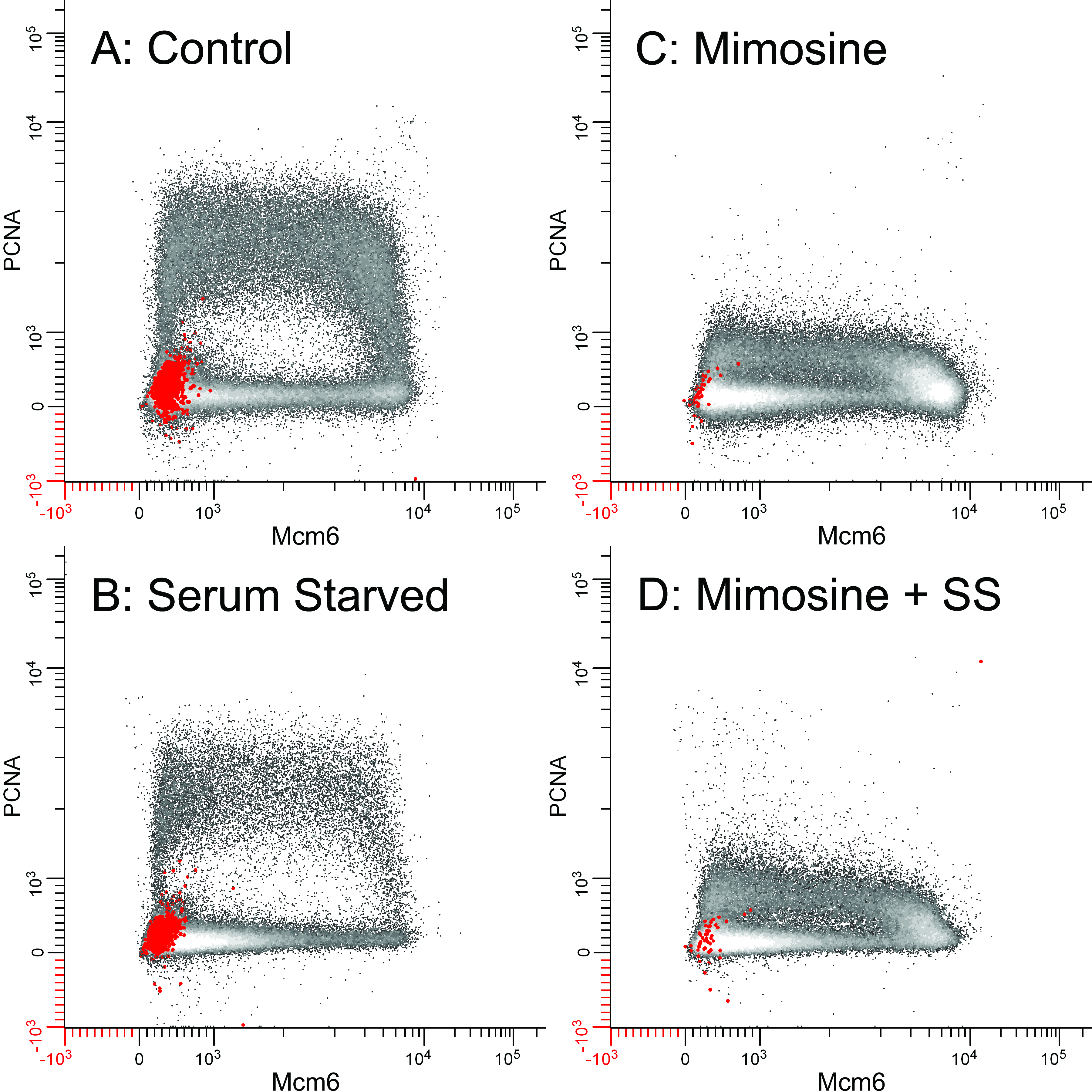

Supplement: Additional file 4 — Mimosine treatment reduces the level of bound PCNA from S phase cells. BJ1 cells were grown in 10% or 0.03% serum in the presence and absence of 120 μg/ml mimosine. After 8 h, treated cells were extracted with Triton X-100 and fixed in methanol (see Methods) before staining for cytometry. Control cells were harvested at time zero. Red dots = mitotic cells. SS = serum starvation. [file 1471-2121-11-26-S4.TIFF]

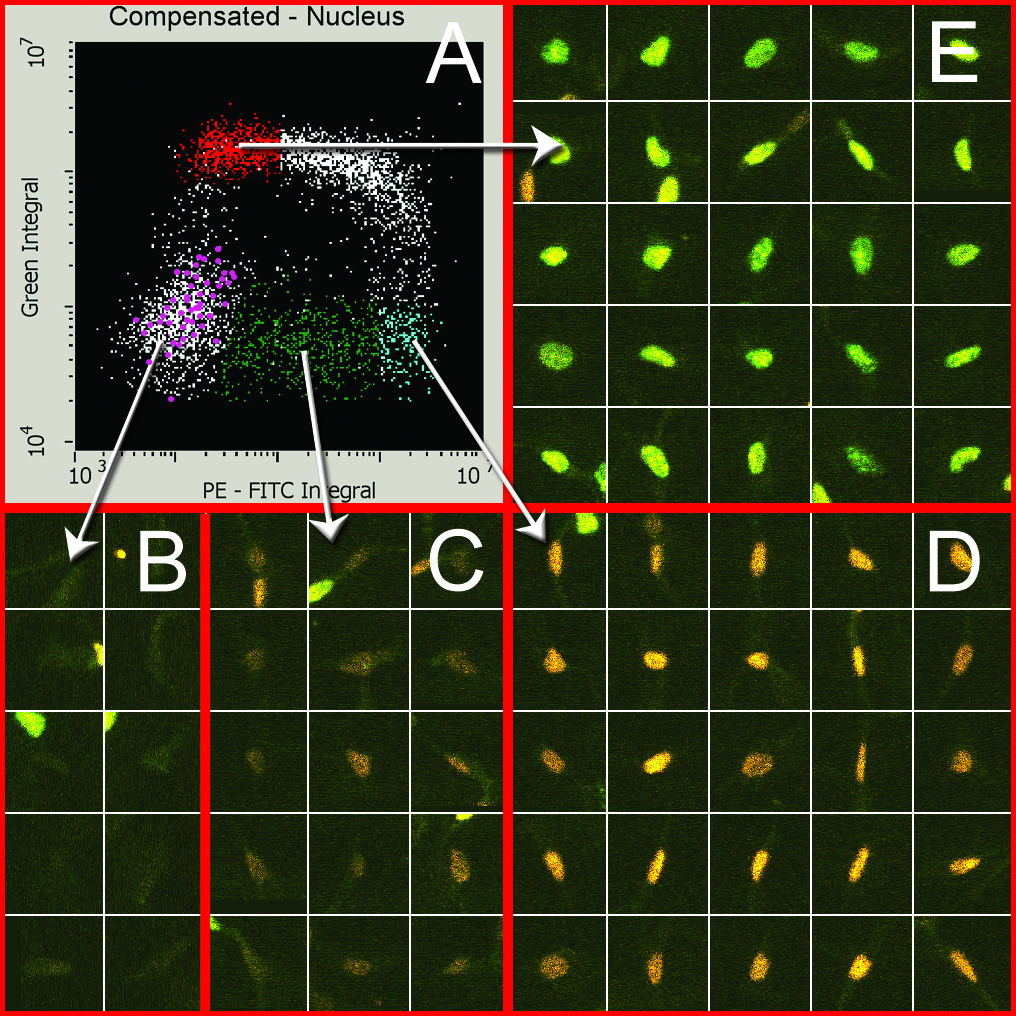

Supplement: Additional file 5 — Laser scanning cytometry. Exponentially growing BJ1 were fixed and stained for Mcm6, PCNA, phospho-S10-histone H3, and DNA as described in Methods. The volume of the staining reaction is greater than flow cytometry samples, but is otherwise the same. These data verify that both signals are nuclear (green = PCNA and orange = Mcm6). Mitotic cells were gated (not shown) and color-coded magenta. The pixels representing the rare mitotic events were made larger in Adobe Photoshop so that they would stand out. These data also support the notion that a more sophisticated analysis of bound Mcm6 versus PCNA might segment G1 into three, rule-based states, G1a1 [G1a cells with the lowest expression of Mcm6 and PCNA, equivalent to mitotic cells] (B); G1a2 [G1a cells (green) expressing Mcm6 at significant but sub-maximal levels] (C), and G1p cells (cyan), defined as the Mcm6 level at which cells enter S (D). Late S phase cells (red) are shown for visual comparison (E). [file 1471-2121-11-26-S5.TIFF]

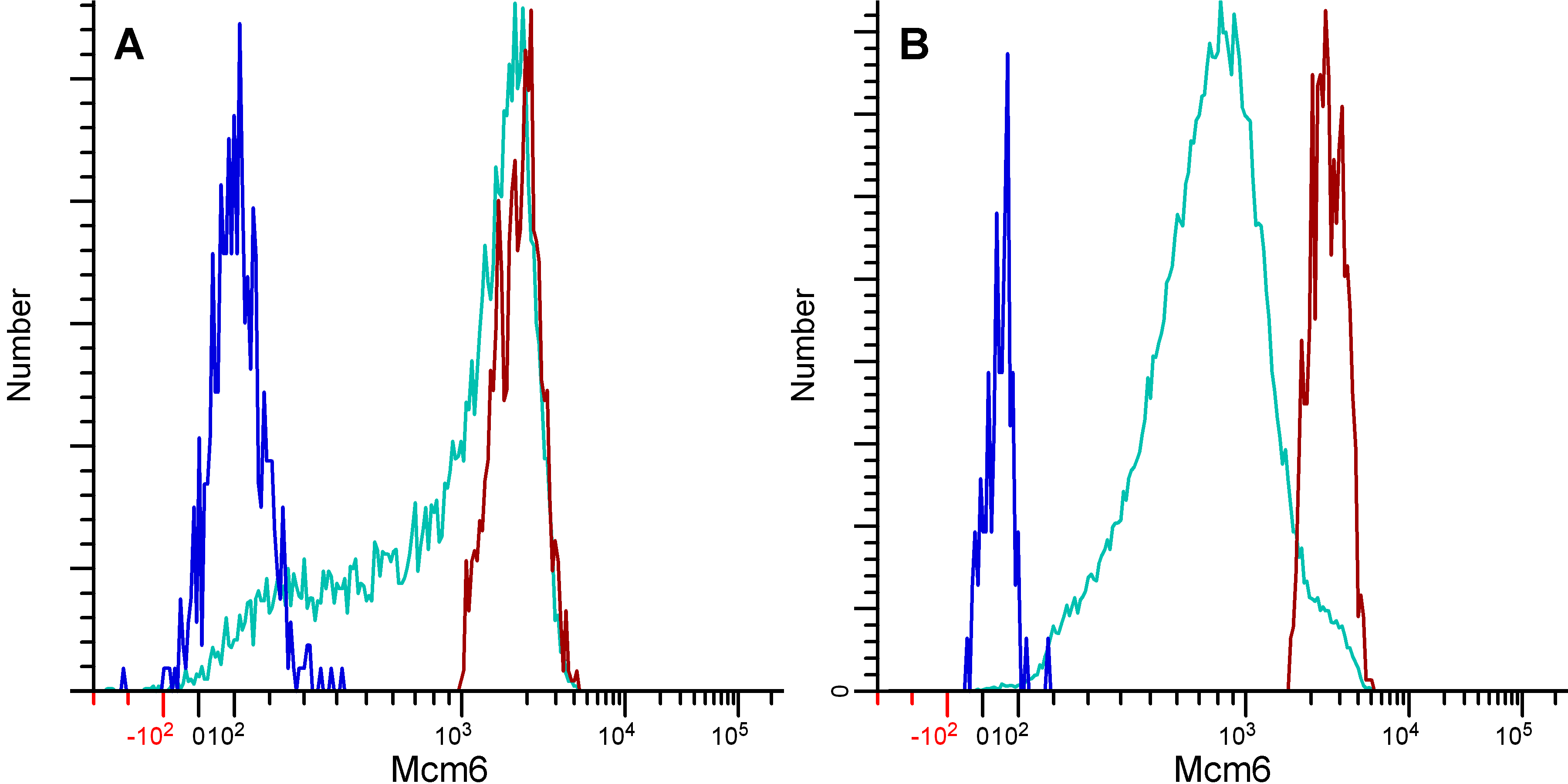

Supplement: Additional file 6 — Flow cytometry of Mcm-6 expressed G1 cells. RPE1 cells were grown exponentially (A) or serum starved for 25 h (B). The cells were fixed then stained for Mcm6, PCNA, phospho-S10-histone H3, and DNA. Mitotic cells (blue histogram) were gated on DNA and phospho-S10-histone H3; early S phase cells (red-brown histogram) were gated on DNA and PCNA, and G1 cells (cyan histogram) were gated on DNA and (absence of) PCNA. The Mcm6 frequency distributions from each gate were overlayed to show the relative expression of each. The distributions are plotted with Y scaling set to the frequency peak height, therefore frequencies are normalized (labeled "number"). The exponentially cycling cells are larger and have higher background fluorescence and therefore, broader coefficients of variation (note the broader mitotic and early S distributions). The key information is that the mitotic levels and S phase levels set the bounds of the distribution (min and max) and that the transition state between them is variably populated with slower growing populations shifted to the left and faster growing populations shifted to the right. [file 1471-2121-11-26-S6.TIFF]
